# Clinical comparison between Multi-Stranded Wires and Single strand Ribbon wires used for lingual fixed retainers

**DOI:** 10.1186/s40510-020-00315-7

**Published:** 2020-06-29

**Authors:** Valiollah Arash, Mehran Teimoorian, Yasamin Farajzadeh Jalali, Sedigheh Sheikhzadeh

**Affiliations:** 1 Department of Orthodontics, School of Dentistry, Babol University of Medical Sciences, Babol, Islamic Republic of Iran; 2 Department of Orthodontics, School of Dentistry, Ilam University of Medical Sciences, Ilam, Islamic Republic of Iran; 3 Dental Materials Research Center, Institute of Health, Babol University of Medical Sciences, Babol, Islamic Republic of Iran

**Keywords:** Fixed orthodontic retainers, Multi-stranded wire, Single-strand ribbon wire, Ligature wire

## Abstract

**Background:**

Long-term retention with fixed retainers with a high success rate seems to be a reasonable solution to minimize or prohibit relapse of orthodontic treatment.

**Methods:**

Two hundred sixty patients between 13 and 30 years old were recruited for this study. The 0.0175 stainless steel twisted wire (G&H Orthodontics, USA) was compared with a single-strand ribbon titanium lingual retainer wire (Retainium, Reliance orthodontics, USA) was used. When treatment was completed, the retainers were bonded from canine to canine in the mandibular arch of the participants. In the follow-up visits, the patients were recalled every 3 months during the 24 months. Detachments, the time of debonding, and side effects were recorded. Statistical analysis was performed by a blinded statistician using a statistical package for Social Science (SPSS, Version20). After descriptive statistics, Kaplan-Meier analysis was performed to measure the survival rates of each retainer. *P* value < 0.05 was considered as significant.

**Results:**

Finally, 138 patients who received twisted wire splint and 112 patients who received ribbon wire were included in the analysis. The average duration of success was about 23 months for twisted wire and ribbon wire, according to the Kaplan-Meier estimates. The analysis showed no significant overall difference between the treatments (*p* = 0.13). Failure rates in terms of detachments in all groups occurred at the enamel junction, and it was 25 in twisted retainer group (18.1%) and was 10 in ribbon retainer group (8.9%); the Kaplan-Meier analysis test detected a significant difference in the failure rates between the groups (*p* = 0/006).

**Conclusions:**

Although the conventional twisted stainless steel wire and single-strand titanium flat metal ribbon wire as fixed orthodontic retainers have the same clinical effects, it was shown that the ribbon wire has less failure in terms of detachments.

## Background

Crowding of lower incisors has a remarkable tendency to relapse [1, 2] regardless of orthodontic techniques [3] and duration [4]. Long-term retention with fixed retainers which is independent of patient’s compliance seems to be a reasonable solution to overcome this problem but unfortunately, an overall bond failure rate of 0.1 to 53% [5, 6] usually at the wire/composite interference had been reported in the literatures [5]. Besides, unexpected complications like torque change between two incisors, opposite inclination of the contralateral canines [6], and space between incisors without any fracture in fixed retainers have also been reported [7].

Different types of mandibular fixed retainer were introduced during the last years. Twenty-eight to 30 mil steel wires were first proposed to fabricate the lingual retainers. Braided steel archwires also seem to be an appropriate choice for splinting lower incisors [8]. Fiber-reinforced composite (FRC) retainers provide an opportunity to minimize the volume of splints and increase aesthetics and can be used in nickel-allergy prone patients [9]. Single-strand titanium flat metal ribbon retainers were introduced recently which seems to be useful to eliminate patient allergy due to its nickel-free nature [10].

Various studies had evaluated the clinical features of FRC and multi-strand stainless steel splints. Although some authors reported no significant difference in bond failure between two types of retainers [7, 11], others found higher bond failure rates in FRC splints [4, 12].

Wire choice may be an important factor to increase the success rate of fixed retainers since some authors claimed that wire fracture decreases in thicker retainer wires [13]. Besides, Cooke claimed that the flexibility of thinner wires may be advantageous to decrease detachment of mandibular lingual retainers [14]. Having both features in metal ribbon retainers may arise a hypothesis that this type may have been a good choice in splinting lower incisors. To our knowledge, no clinical follow-up study had compared the bond failure of ribbon metal retainers and multi-stranded stainless steel splints, so the aim of this study was to assess the bond failure rate of these two types of retainers through a prospective study.

## Methods

The Ethics Committee of Babol University of Medical Sciences approved the present study (letter number: IR.MUBABOL.REC.1398.024).

In this prospective study, a total of 260 patients between 13 and 30 years old in the finishing phase of orthodontic treatment were selected. The patients had finished their fixed orthodontic treatment with 0.022 in. MBT bracket system in a private orthodontic office. These patients had final class I occlusion with good oral hygiene. Patients with a deep overbite or any sign of periodontal problems like bleeding on probing or pocket depth more than 3 mm were excluded from the study. The patients were randomly divided into two groups. Lower anterior teeth were splinted using 0.0175 stainless steel twisted wire (G&H Orthodontics, USA) in the first group. In the second group, single-strand ribbon titanium lingual retainer wire (Retainium, Reliance Orthodontics, USA) was used. The fixed retainer wires were formed by an expert orthodontist on the final working casts.

After scaling and polishing of dental arches, lip retractor and cotton rolls were used to prevent moisture contamination. The lingual surface of six lower teeth was etched by 37% of phosphoric acid gel (3M Unitek, Monrovia, USA) for 30 s. After rinsing with water and air drying, the retainers were fixed with dental floss and a thin layer of bonding primer (Transbond XT, 3M Unitek, Monrovia, USA) was used. Then, composite resins were light-cured and polished carefully.

The patients were recalled every 3 months during a 24-month follow-up period. Detachments, time of debonding, and side effects were recorded.

Statistical analysis was performed by a blinded statistician using a statistical package for Social Science (SPSS, Version20). After descriptive statistics, Kaplan-Meier analysis was performed to measure the survival rates of each retainer. *P* value < 0.05 was considered significant.

## Results

Two hundred sixty patients were included in this study. Ten participants dropped out of the study because of failure to regularly attend the follow-up sessions. Finally, 138 patients who received twisted wire splint and 112 patients who received ribbon wire were included in the analysis. The trial ended after 24 months of follow-up.

The mean age of the included patients was 20 ± 4.35 years old and twisted wire 21.08 ± 4.09, and the ribbon wire was 19.70 ± 4.68. The average ages were similar between the three groups, according to the ANOVA (*p* = 0.04). A ribbon wire splint was used for 44 men and 68 women, and a twisted wire retainer was used for 55 men and 83 women. The difference between the gender distributions of the first group (55 males and 83 females) and second group (44 males and 68 females) was not significant (chi-squared *p* = 0.92) (Table 1).

**Table 1:** Descriptive data of patients participated in this study

Variable		Twisted wire	Ribbon Wire	*P* value
Sex	Male	55 (39.99%)	44 (39.3%)	0.92
	Female	83 (60.1%)	68 (60.7%)	
Age (mean ± SD)		21.08 ± 4.09	19.70 ± 4.68	0.04

### Failure rates

Failure rates in terms of detachments in all groups seem to have occurred at the enamel junction which is clinically observed the bulk of detached composite, and it was 25 in twisted retainer group (18.1%) and was 10 in ribbon retainer group (8.9%); the Kaplan-Meier analysis test detected a significant difference in the failure rates between the group (*p* = 0.006) (Table 2).

**Table 2:** Survival time statistics calculated using the Kaplan-Meier analysis

Treatment	Success	Fail	Mean failure time (month)	95% CI	
Twisted wire	113 (81.9%)	25 (18.1%)	23.48	23.14	23.81
Ribbon wire	102 (91.1%)	10 (8.9%)	23.53	23.09	23.97

*CI* confidence interval

### Duration of successful retainer use

**Fig. 1:**
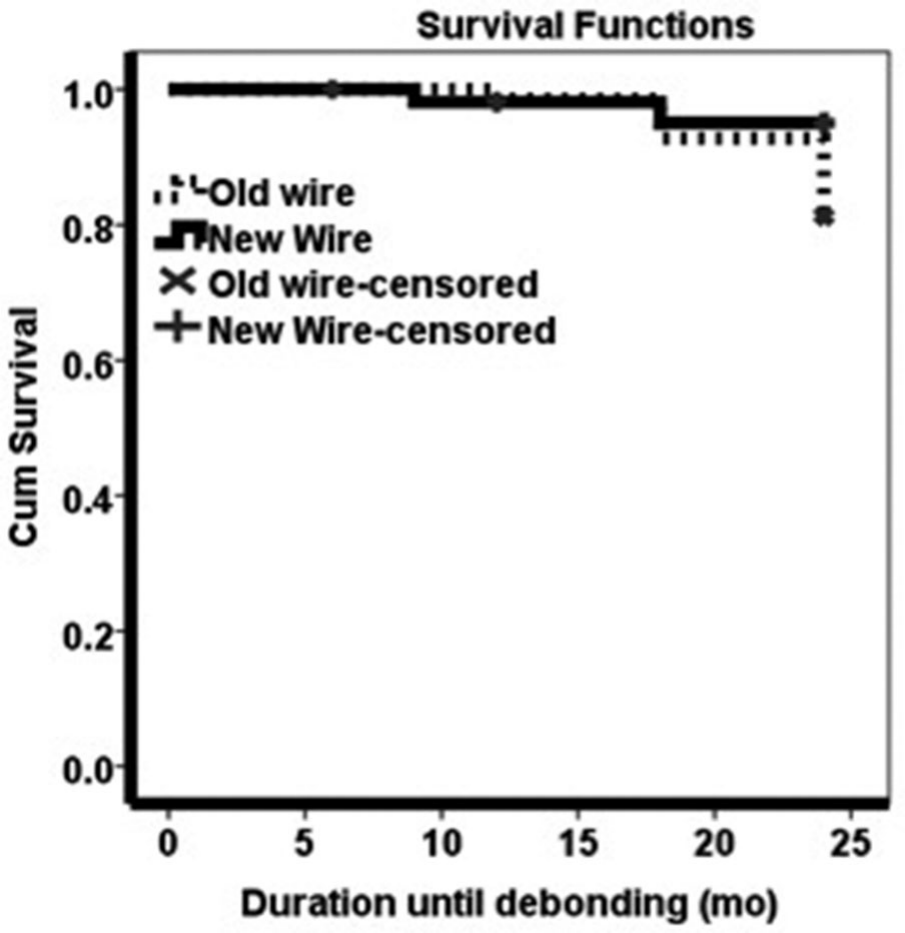
Comparing duration of successful between two retainer groups

The average duration of success was about 23 months for twisted wire and ribbon wire, according to the Kaplan-Meier estimates (Fig. 1). The analysis showed no significant overall difference between the treatments (*p* = 0.13) (Table 3).

**Table 3:** The results of twisted wire with the ribbon wire as the reference

Treatment	P value	95% CI for HR	
Twisted wire	0.014	1.313	11.110
Ribbon wire	0.006	1.459	9.891

*CI* confidence interval

The analysis showed 3.798 times risk of failure (*p* = 0.006), and in the adjusted state, the risk of failure was more than 3.819 times higher in the twisted retainers compared to ribbon retainers (*p* = 0.014) (Table 4).

**Table 4:** The results of the Cox regression, comparing the old wire with the new wire as the reference

Treatment	*P* value	95% CI for HR	
Twisted wire	0.014	1.313	11.110
Ribbon wire	0.006	1.459	9.891

*CI* confidence interval

## Discussion

Splinting the teeth after orthodontic treatment is a common clinical procedure. Multi-strand wires appear to be the most popular for direct-bonded retainers, and its retentive efficacy and reliability have been proved [13]. In this study, splinting the teeth with ribbon metal wire that has gained popularity in the last years was compared. The results showed that the reliability with the multi-stranded wire retainer was comparable to the ribbon retainer, and there was no significant overall difference between the treatments of old (twisted wire) and new (ribbon wire) retainers (*p* = 0.13).

The duration of the success for the multi-strand wire was about 23 months that was not significantly different from the ribbon wire. Rose et al. showed a similar survival time for multi-strand wire. They concluded that in terms of reliability, the direct-bonded multi-strand wire is superior to the plasma-treated polyethylene woven ribbon and resin retainer [12]. Similar findings have been described for glass fiber reinforced retainers [15]. In contrast with these studies, Scribante et al. showed no statistically significant differences in survival time after 12 months for the multi-strand wire and FRC. They described that the use of different materials and different bonding techniques could be the reason for different results [11].

The study of Sobouti et al. was about a 2-year survival analysis of twisted wire-fixed retainer versus spiral wire and fiber-reinforced composite retainers [14]. Although the failure rate of the twisted wire retainer was two times lower than that of the FRC retainer, the differences between the survival rates were not significant. FRC retainers might have a higher failure rate because of their lower flexibility, which results in higher strain in the inter-dental areas under loading. Among different FRC retainers, the ribbon type displayed the highest bond strength. Salehi et al. showed that the mean survival time and the rate of broken or detached ribbon retainers and multi-strand retainers are comparable [4].

Against this result, the present study showed that the failure rate of the ribbon metal wire was more than two times lower than that of the twisted wire. The failure rates in the present study were considered in terms of detachments; but it is better to consider that the failure should be evaluated with regard to not only the adhesion quality but also the clinical reversibility, with the least damage to enamel during removal or repair of the failed retainer. So, the results based on the method of the study might vary in different studies. In the study of Foek et al., the ribbon retainers presented adhesive failure and material breakage in 50% and 40% of the specimens, respectively [16].

Foek and his colleagues evaluated fatigue resistance, debonding force, and failure type of fiber-reinforced composite, polyethylene ribbon-reinforced, and braided stainless steel wire lingual retainers and stated that the retainers presented similar debonding forces, but different failure types and braided stainless steel wire retainers presented the most repairable failure type [16]. The difference might be related to types of studies because we performed a clinical trial but the study of Foek et al. was in vitro.

In a systematic review that was conducted by Iliadi et al., the failure of fixed orthodontic retainers was evaluated. The random-effects meta-analysis between two studies that compared polyethylene woven ribbon vs multi-stranded wire retainers indicated no statistically significant difference in the risk of failure between the treatment groups [17].

Finally, it should be noted that although fixed orthodontic retainers have been used in clinical practice for many years, the best protocol for post orthodontic treatment still remains an important issue. Iliadi et al. also mentioned in their systematic review that the available studies and their hypothesis cannot provide reliable evidence about fixed orthodontic retainers. They concluded that despite the numerous studies dealing with parameters of fixed retention, there is a lack of evidence on the selection of the optimal protocol and materials for that [17].

## Conclusion

Although the conventional twisted stainless steel wire and single-strand titanium flat metal ribbon wire as fixed orthodontic retainers have the same clinical effects, it was shown that the ribbon wire has less failure in terms of detachments.

## Data Availability

The datasets used and analyzed during the current study are available from the corresponding author on reasonable request.

## Abbreviations

FRC: Fiber-reinforced composites
